# Overexpression of a brassinosteroid biosynthetic gene *Dwarf* enhances photosynthetic capacity through activation of Calvin cycle enzymes in tomato

**DOI:** 10.1186/s12870-016-0715-6

**Published:** 2016-01-28

**Authors:** Xiao-Jing Li, Xie Guo, Yan-Hong Zhou, Kai Shi, Jie Zhou, Jing-Quan Yu, Xiao-Jian Xia

**Affiliations:** Department of Horticulture, Zijingang Campus, Zhejiang University, Hangzhou, 310058 P.R. China; Zhejiang Provincial Key Laboratory of Horticultural Plant Integrative Biology, Hangzhou, 310058 China

**Keywords:** Brassinosteroids, 2-cystein peroxiredoxins, *Dwarf*, Glutathione, Photosynthesis, RuBisCO

## Abstract

**Background:**

Genetic manipulation of brassinosteroid (BR) biosynthesis or signaling is a promising strategy to improve crop yield and quality. However, the relationships between the BR-promoted growth and photosynthesis and the exact mechanism of BR-regulated photosynthetic capacity are not clear. Here, we generated transgenic tomato plants by overexpressing *Dwarf*, a BR biosynthetic gene that encodes the CYP85A1, and compared the photosynthetic capacity with the BR biosynthetic mutant *d*^*im*^ and wild type.

**Results:**

Overexpression of *Dwarf* promoted net photosynthetic rate (*P*_N_), whereas BR deficiency in *d*^*im*^ led to a significant inhibition in *P*_N_ as compared with WT. The activation status of RuBisCO, and the protein content and activity of RuBisCO activase, but not the total content and transcripts of RuBisCO were closely related to the endogenous BR levels in different genotypes. However, endogenous BR positively regulated the expression and activity of fructose-1,6-bisphosphatase. *Dwarf* overexpression enhanced the activity of dehydroascorbate reductase and glutathione reductase, leading to a reduced redox status, whereas BR deficiency had the contrasting effects. In addition, BR induced a reduction of 2-cystein peroxiredoxin without altering the protein content.

**Conclusions:**

BR plays a role in the regulation of photosynthesis. BR can increase the photosynthetic capacity by inducing a reduced redox status that maintains the activation states of Calvin cycle enzymes.

**Electronic supplementary material:**

The online version of this article (doi:10.1186/s12870-016-0715-6) contains supplementary material, which is available to authorized users.

## Background

Photosynthesis is the fundamental basis for the biomass accumulation in plants. Sugars derived from photosynthesis provide the building blocks for the growth of plants. Moreover, sugars act as signals that interconnect with hormonal signaling pathways, leading to the reprogramming of gene expression and activation of a wide range of developmental processes [1–3]. Recent studies have shown that photosynthesis is a major limiting factor for improving the yield potential of crops [4]. However, the photosynthetic rates of cultivated crops are far below optimum values predicted by the theoretical models [4]. Photosynthesis is limited by the sink demand for carbohydrates, the efficiency of carboxylation of ribulose bisphosphate (RuBP) carboxylase/oxygenase (RuBisCO), the quantum yield of photochemical reactions, and the CO_2_ diffusion through the stomata and mesophyll cells [5]. In addition, environment stresses adversely affect photosynthesis of higher plants in their natural habitat. Therefore, understanding the mechanisms involved in the regulation of photosynthetic processes is of vital importance to optimize CO_2_ assimilation and improve the yield potential of crops.

A large body of evidence has shown that photosynthetic capacity is influenced by hormone homeostasis or signaling in plants. Low levels of ethylene promote plant growth [6], whereas ethylene insensitivity results in down-regulation of RuBisCO expression and photosynthetic capacity [7]. Alterations in the expression of genes involved in gibberellin (GA) biosynthesis and inactivation results in increased and decreased biomass accumulation, respectively, which is positively correlated with the photosynthetic rates at the whole plant level [8]. In addition, GA plays a positive role in enhancing the photosynthetic activity, at least partly by regulating the development of chloroplasts [9]. Abscisic acid (ABA) is a well-known regulator of stomatal closure, which decreases photosynthesis by increasing the limitation of CO_2_ diffusion [10]. Furthermore, ABA is required for the maintenance of the photochemical quenching and acclimation to abiotic stresses [11]. However, our knowledge on the mechanisms of hormone-mediated regulation of photosynthesis is still fragmentary.

Brassinosteorids (BRs) are a group of steroids in plants [12]. BRs are involved in virtually all the developmental process in a plant’s life [13]. The identifications of BR biosynthetic and signaling mutants have dissected the roles of BRs as plant hormones and paved the way for revealing the mechanism of BR signaling [14]. Despite a detailed understanding of the BR signaling pathway, it is not clear how exactly BRs control growth. Characterization of transgenic rice overexpressing BR biosynthetic gene revealed that BR regulates the grain filling and increases the yield [15]. The rice *small grain1* mutant, which contains a mutation in mitogen-activated protein kinase kinase 4, shows defects in cell proliferation and reduced response to BR [16]. Previous study has shown that BR promotes cell division through transcriptional regulation of cyclin D [17]. However, the relationship between BR-promoted growth and photosynthesis is not clear.

The yield increment in transgenic rice overexpressing BR biosynthetic gene is associated with increased CO_2_ assimilation rates and enhanced assimilates flow to the grains [15]. Antisense inhibition of *CPD* gene, which is critical for BR biosynthesis, leads to reduction of CO_2_ assimilation [18]. However, it is not clear whether the reduced photosynthetic capacity in the transgenic lines resulted from the altered carbohydrate metabolism, reduced quantum yield of photochemical reaction, or the inhibition of Calvin cycle. By using exogenous BR or its biosynthesis inhibitor, we found that BR plays a vital role in the regulation of photosynthesis by activating the RuBisCO [19, 20]. However, the genetic evidence is still lacking. In this study, we generated transgenic tomato plants overexpressing the BR biosynthetic gene *Dwarf*, which encodes a CYP85A1, and compared the photosynthetic capacity of the transgenic lines and BR biosynthesis mutant with the wild type. We found a close relationship between BR levels of the plants and the photosynthetic capacity. BR-promoted photosynthetic capacity was associated with the enhanced antioxidant capacity and a reduced cellular redox status. The potential mechanism by which BR promotes photosynthesis through redox regulation of Calvin cycle enzymes was proposed.

## Methods

### Transformation and plant growth conditions

The *Dwarf* gene was PCR amplified from the cDNA of tomato (*Solanum lycopersicum* L. cv Condine Red) using forward primer 5’-GGGGTACCCCATGGCCTTCTTC-3’ and reverse primer 5’-GCTCTAGAGCTTAGTGAGCTGAAAC-3’ based on the published sequence (Sol genomic network accession Solyc02g089160.2). For transformation, we used the binary vector pMV2, which carries the spectinomycin resistance gene for bacterial selection and the neomycin phosphotransferase II gene for selection of transformed plants [21]. The binary vector was constructed by inserting the *Dwarf* cDNA between the KpnI and XbaI sites in the sense orientation driven by the cauliflower mosaic virus 35S promoter. *Agrobacterium tumefaciens* strain C58 was used to mediate introducing the vector into callus of tomato cultivar Condine Red. After screening for regenerated shoots on selection medium containing kanamycin, the transgenic plants were further verified by PCR using genomic DNA as template and 35S forward and gene-specific reverse primers. Two lines (DWF:OX2, and DWF:OX3) were chosen for the experiments.

To compare the photosynthetic capacity, wild type Condine Red (CR), the BR biosynthesis mutant *d*^*im*^ (in the background of CR), and DWF:OX2 and DWF:OX3 were used. The *d*^*im*^ mutant was obtained from the Tomato Genetics Resource Center (University of California, Davis, CA, USA, accession LA0571). These seeds were germinated and grown in a mixture of peat and vermiculite (1:1, v/v) under a 16 h light (200 μmol m^−2^ s^−1^; at 25 °C) and 8 h dark (at 20 °C) cycle. Two-month-old plants were used for the experiments. For assay of Calvin cycle enzyme activity, leaf discs were harvested from different genotypes, frozen immediately in liquid nitrogen, and stored at -80 °C prior to analysis. For activity of antioxidant enzymes, the samples were collected based on fresh weight, whereas for gene expression, the whole leaflets were collected.

### Leaf gas exchange and chlorophyll fluorescence analysis

Gas exchange analysis was conducted on the 6^th^ leaf from tomato plants using an open gas exchange system (LI-6400; LI-COR, Lincoln, NE, USA). The measurements were taken from 8 to 11 am in the morning. The assimilation versus intercellular CO_2_ concentration (A/Ci) curve was determined according to von Caemmerer and Farquhar [22]. The maximum ribulose-1,5-bisphosphate carboxylase/oxygenase (RuBisCO) carboxylation rates (*V*_c,max_) and maximum ribulose-1,5-bisphosphate (RuBP) regeneration rates (*J*_max_) were estimated from the A/Ci curves using the method described by Ethier and Livingston [23].

Chlorophyll fluorescence parameters were determined by analyzing the slow kinetics curve using Dual-PAM-100 system (Walz, Germany). The analysis started after the plants were dark-adapted for 30 min. The initial fluorescence (Fo) was obtained after switching on the measuring beam, and then the maximum fluorescence (Fm) was obtained after applying a 0.8 s saturating pulse (>10000 μmol m^−2^ s^−1^). After the fluorescence signal decayed for 20s, the actinic light (280 μmol m^−2^ s^−1^) was switched on for 300 s, during which the saturating pulse was applied every 20s. The steady state fluorescence (Fs) and maximum fluorescence under illumination (Fm’) were recorded before and during the saturating pulse, respectively. The minimal fluorescence under illumination (Fo’) was calculated according to Baker [24]. Maximum quantum yield of PSII (Fv/Fm), actual quantum yield of PSII (Ф_PSII_), efficiency of antenna excitation transfer (Fv’/Fm’), and photochemical quenching coefficient (qP) were calculated as (Fm-Fo)/Fm, (Fm’-Fs)/Fm’, (Fm’-Fo’)/Fm’, and (Fm’-Fs)/(Fm’-Fo’), respectively.

### Measurement of endogenous BRs, total chlorophyll and soluble protein contents

Endogenous BRs were determined using solid-phase extraction with double-layered cartridge followed by high-performance liquid chromatography–tandem mass spectrometry [25]. For the determination of BRs and their metabolites, (^2^H_3_) castasterone (D-Cs) and (^2^H_3_) brassinolide (D-BL) were spiked into the extraction solution with 1 g of leaf sample. Total chlorophyll content was determined by the method of Arnon [26]. Total soluble protein content was measured using Bradford reagent (Bio-Rad, Hercules, California).

### Western-blot analysis of rbcL, rbcS and RCA

Proteins were extracted from leaf samples as described previously [20]. For Western-blot analysis, proteins were separated by SDS-PAGE using 12.5 % (w/v) acrylamide gels and electrophoretically transferred to nitrocellulose membranes (Millipore, Saint-Quentin, France). The proteins were detected with commercial antibodies raised against rbcL, rbcS and RCA (Agrisera, Vannes, Sweden).

### Non-reducing SDS-PAGE and western blot analysis of 2-cystein peroxiredoxin

For detection of 2-cystein peroxiredoxin (2-CP), the samples were extracted with buffer containing 100 mM HEPES, pH 7.5, 5 mM EDTA, 5 mM EGTA, 10 mM Na_3_VO4, 10 mM NaF, 50 mM β-glycerophosphate, 1 mM phenylmethylsulphonyl fluoride, 10 % glycerol, 7.5 % polyvinylpolypyrrolidone (PVP), 10 mM dithiothreitol (DTT) and 10 mM N-ethylmaleimide (NEM, thiol-blocking reagent). The grinded samples were centrifuged at 13 000 g for 20 min. For analysis of the redox status of 2-CP, DTT was omitted in the extraction buffer as described previously [27]. Protein samples (15 μg) supplemented with 5× loading buffer [225 mM Tris–HCl, pH 6.8, 5 % (w/v) SDS, 50 % glycerol, 0.05 % bromophenol blue] were separated via 12 % SDS-PAGE, and 2-CP was detected through western blot as described previously [28] with a polyclonal antibody against 2-CP (Beijing Protein Innovation, Beijing, China).

### Determination of RuBisCO, RuBisCO activase (RCA), and FBPase activity

RuBisCO activity was measured spectrophotometrically by coupling 3-phosphoglyceric acid formation with NADH oxidation according to Ward and Keys [29] with some modifications. Leaf discs were homogenized with extraction buffer (50 mM HEPES, pH 8.0, 1 mM EDTA, 10 mM MgCl_2_, 2 % insoluble PVPP and 10 mM β-mercaptoethanol). The homogenate was centrifuged at 4 °C for 15 min at 15000 g. Total activity was assayed after the crude extract had been activated in a 0.1 ml activation mixture containing 50 mM HEPES (pH 8.0), 26.6 mM MgCl_2_ and 16.6 mM NaHCO_3_ for 15 min at 28 °C. The measurement of initial RuBisCO activity were carried out in 0.1 ml of reaction medium containing 50 mM HEPES-NaOH (pH 8.0), 10 mM NaHCO_3_, 20 mM MgCl_2_, 1 U creatine phosphokinase, 1 U 3-phosphoglyceric phosphokinase, 1 U glyceraldehydes 3-phosphate dehydrogenase, 0.5 mM ATP, 0.015 mM NADH, 0.5 mM phosphocreatine and 0.06 mM RuBP. The change in absorbance at 340 nm was monitored for 90 s. RCA activity was determined using a RuBisCO Activase Assay Kit (Genmed Scientifics, Washington, DC, USA). Briefly, 0.2 g leaf sample was rapidly ground in a 15-mL tube with liquid N_2_. Then, 500 μL lysis buffer was added. The mixture was vortexed and homogenized. The homogenate was transferred to a 1.5-mL eppendorf tube and centrifuged at 4 °C for 5 min at 300 g. Aliquot of supernatant was transferred to a new eppendorf tube and centrifuged at 4 °C for 10 min at 1000 g. The supernatant was removed and the pellet was suspended with 200 μL lysis buffer. The suspension was used for RCA activity assay. RCA activity was measured based on RuBisCO activity after incubating inactivated RuBisCO with enzyme extract in the presence or absence of ATP. RuBisCO activity was measured by the coupled spectrophotometric assay.

FBPase activity was determined by monitoring the absorption at 340 nm, using an extinction coefficient of 6.2 mM^−1^ cm^−1^ [30]. Total activity was assayed after the crude extract had been activated in a 0.1 ml activation mixture containing 100 mM DTT, 2 mM fructose-1,6-bisphosphate (FBP), 10 mM MgCl_2_, and 0.1 M Tris–HCl (pH 8.0). The initial activity was assayed immediately after homogenization. The assay mixture consisted of 0.1 M HEPES (pH 8.0), 0.5 mM Na_2_EDTA, 10 mM MgCl_2_, 0.3 mM NADP^+^, 0.6 mM FBP, 0.6U of glucose-6-phosphate dehydrogenase from baker’s yeast (Sigma, Santa Clara, CA, USA), 1.2U of glucose phosphate isomerase from baker’s yeast (Sigma, Santa Clara, CA, USA), and 100 μl of enzyme extract in a final volume of 1 ml.

### Measurements of glutathione and ascorbate contents

For the measurement of reduced glutathione (GSH) and oxidized glutathione (GSSG), plant leaf tissue (0.3 g) was homogenized in 2 ml of 6 % metaphosphoric acid containing 2 mM EDTA and centrifuged at 4 °C for 10 min at 12 000 g. After neutralization with 0.5 M phosphate buffer (pH 7.5), 0.1 ml of the supernatant was added to a reaction mixture containing 0.2 mM NADPH, 100 mM phosphate buffer (pH 7.5), 5 mM EDTA, and 0.6 mM 5,5’-dithio-bis (2-nitrobenzoic acid). The reaction was initiated by adding 3U of glutathione reductase (GR) from yeast (Sigma, Santa Clara, CA, USA) and was monitored by measuring the changes in absorbance at 412 nm for 1 min. For the GSSG assay, GSH was masked by the addition of 40 μl of 2-vinylpyridine to the neutralized supernatant, whereas 40 μl of water was added for the total glutathione assay. The GSH concentration was obtained by subtracting the GSSG concentration from the total concentration [31].

### Determination of the activity of enzymes involved in the AsA-GSH cycle

To determine the activities of enzymes involved in the AsA-GSH cycle, leaf tissue (0.3 g) was ground in 3 ml of ice-cold buffer containing 25 mM HEPES (pH 7.8), 0.2 mM EDTA, 2 mM ascorbic acid, and 2 % PVP. The homogenates were centrifuged at 4 °C for 20 min at 12 000 g, and the resulting supernatants were used to determine the enzymatic activity. The dehydroascorbate reductase (DHAR) activities were evaluated by measuring the increase in absorbance at 265 nm, as described by Nakano and Asada [32]. GR activity was measured according to the decrease of absorbance at 340 nm based on the method described by Halliwell and Foyer [33]. All spectrophotometric analyses were conducted in a SHIMADZU UV-2410PC spectrophotometer (Shimadzu Corporation, Kyodo, Japan).

### Total RNA extraction and gene expression analysis

Total RNA was isolated from tomato leaves using the TRIZOL reagent (Sangon, Shanghai, China) according to the instructions supplied by the manufacturer. After extraction, the total RNA was dissolved in RNase-free water. The cDNA template for qRT-PCR was synthesized from 2 μg of total RNA using the ReverTra Ace qPCR RT Kit (Toyobo, Osaka, Japan).

For qRT-PCR analysis, PCR products were amplified using iQ SYBR Green SuperMix (Bio-Rad, Hercules, CA, USA) in 25 μl assays. PCR was performed using the iCycleriQ 96-well real-time PCR Detection System (Bio-Rad, Hercules, CA, USA), and the cycling conditions consisted of denaturation at 95 °C for 3 min, followed by 40 cycles of denaturation at 95 °C for 30 s, annealing at 58 °C for 30 s, and extension at 72 °C for 30 s. The tomato *actin* gene was used as an internal control. Primers used for the qRT-PCR analysis were listed in the Additional file 1: Table S1. Relative gene expression was calculated as described by Livak and Schmittgen [34].

### Statistical analysis

The experimental design was a completely randomized block design with four replicates. Each replicate contained ten plants. Statistical analysis were performed by SPSS statistical software (ver.19.0, SPSS Inc., Chicago, IL, USA), using one-way analysis of variance (ANOVA). To evaluate the treatment effects, a Tukey’s test (*P* < 0.05) was performed.

## Results

### BRs promoted CO_2_ assimilation in tomato plants

**Fig. 1:**
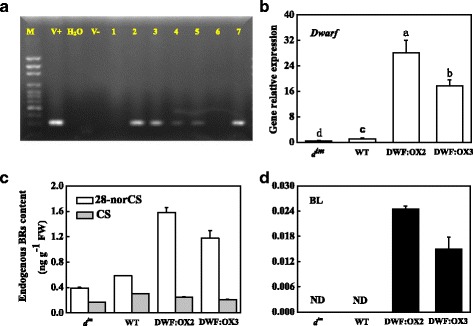
BR biosynthesis capacity in mutant defective in *Dwarf* (*d* ^*im*^), wild type (WT) and *Dwarf* overexpressing lines (DWF:OX2 and DWF:OX3). **a** Confirmation for the presence of transgene in the selected lines by PCR. M, 100 bp marker; V+, transformed vector; V-, genomic DNA of WT plants; 1–7, selected plant lines. **b**–**d** *Dwarf* transcript and accumulation of BR biosynthetic precursors. Leaves were harvested at 45d after germination for the gene expression and BRs content analysis. Data are the means ± SD of four independent biological samples. Means denoted by the same letter did not differ significantly according to Tukey’s test (*P* < 0.05). CS, castasterone; 28-norCS, 28-norcastasterone; BL, brassinolide

To examine the role of endogenous BRs in the regulation of photosynthesis, we overexpressed *Dwarf* gene encoding the CYP85A1 that catalyzes the conversion of 6-deoxocastasterone to castasterone in tomato plants and obtained several independent transgenic lines (Fig. 1). The presence of 35S promoters in the transgenic lines was confirmed by PCR. Considering the expression level of the transgene, two lines, DWF:OX2 and DWF:OX3 were selected for further study. qPCR analysis indicated that the expression of *Dwarf* was enhanced by more than 28 and 17 folds in DWF:OX2 and DWF:OX3, respectively, whereas it was suppressed by *c.a.* 60 % in the *d*^*im*^ mutant as compared to the wild type (WT). Analysis of BR biosynthesis precursors showed that the content of 28-norCS (28-norcastasterone) was significantly increased whereas the content of (castasterone) was slightly decreased in the transgenic lines. In contrast, the content of both 28-norCS and CS decreased in *d*^*im*^ mutant. In our study, BL (brassinolide) was undetectable in both *d*^*im*^ and WT plants but detectable in both *Dwarf* overexpressing lines.

**Fig. 2:**
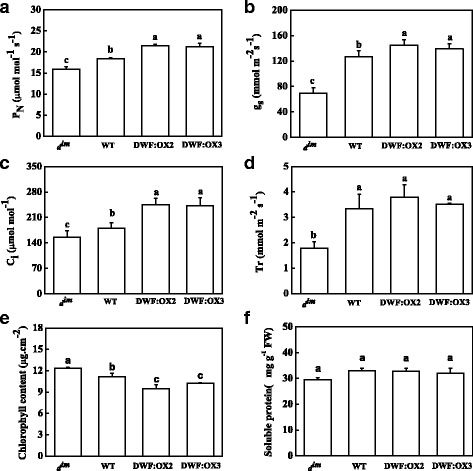
Effects of *Dwarf* gene overexpression (DWF:OX2 and DWF:OX3) and mutation (*d* ^*im*^) on net photosynthetic rates (*P*_N_), (**a**), stomatal conductance (g_s_), (**b**), intercellular CO_2_ concentration (C_i_), (**c**), transpiration rate (Tr), (**d**), total chlorophyll content (**e**) and soluble protein content (**f**). Data are the means of four replicates with SDs. Means followed by the same letter are not significantly different according to Tukey’s test (*P* < 0.05)

To understand the requirement of *Dwarf* for photosynthesis, we first compared the gas exchange parameters among *d*^*im*^, WT and the *Dwarf* overexpressing lines. Results showed that defects in BR biosynthesis significantly inhibited net photosynthetic rates (*P*_N_) in *d*^*im*^ as compared to WT (Fig. 2a). Similarly, the stomatal conductance (g_s_), intercellular CO_2_ concentration (C_i_) and transpiration rate (Tr) were significantly decreased in *d*^*im*^ mutant. Meanwhile, compared with WT plants, the *P*_N_ and C_i_ were significant increased in DWF:OX2 and DWF:OX3. However, the g_s_ and Tr were not significantly affected (Fig. 2b, c and d). The total chlorophyll content of *d*^*im*^ mutant was significantly increased, while that of transgenic lines was significantly decreased as compared to WT (Fig. 2e), indicating that the promotion of photosynthesis by BR was not related to the chlorophyll and/or light absorption capacity. Total soluble protein content showed no difference among *d*^*im*^, WT and the *Dwarf* overexpressing lines (Fig. 2f), suggesting that BR-promoted photosynthesis is probably not associated with nitrogen assimilation.

### BRs increased photochemical quantum yield and in vivo RuBP carboxylation and regeneration capacity

**Fig. 3:**
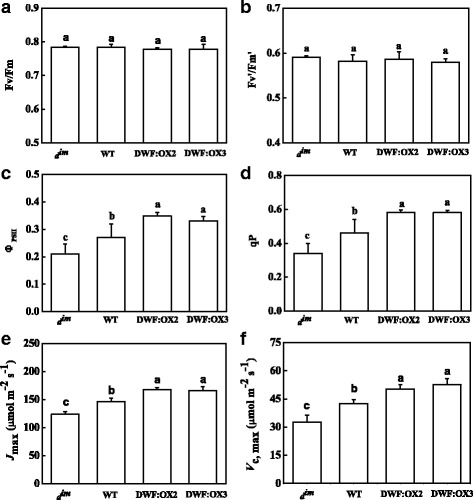
Effects of *Dwarf* gene overexpression (DWF:OX2 and DWF:OX3) and mutation (*d* ^*im*^) on maximum quantum yield of PSII (Fv/Fm), (**a**), antenna excitation transfer efficiency (Fv’/Fm’), (**b**), actual quantum yield of PSII (Ф_PSII_), (**c**), photochemical quenching coefficient (qP), (**d**), maximum ribulose-1,5-bisphosphate (RuBP) regeneration rates (*J*_max_), (**e**), and maximum ribulose-1,5-bisphosphate carboxylase/oxygenase (RuBisCO) carboxylation rates (*V*_c,max_), (**f**). Data are the means of four replicates with SDs. Means followed by the same letter are not significantly different according to Tukey’s test (*P* < 0.05)

To further examine the mechanism by which BR regulates photosynthesis, we analyzed the chlorophyll fluorescence, which indicated the functional status of photosystem II (PSII). The results showed that the maximum quantum yield of PSII (Fv/Fm), which reflects the photoinhibition to the PSII, was not affected by endogenous BR levels in different genotypes at least in normal condition in this study (Fig. 3a). Similarly, the antenna excitation transfer efficiency (Fv’/Fm’) was not affected by the BR levels, indicating that BR levels had no effects on the energy dissipation in the antenna of PSII (Fig. 3b). Consistent with the changes in *P*_N_, the actual quantum yield of PSII (Ф_PSII_) was inhibited in *d*^*im*^, whereas Ф_PSII_ was significantly increased in DWF:OX2 and DWF:OX3 as compared to WT (Fig. 3c). Changes in photochemical quenching coefficient (qP), which reflects the fraction of PSII being able to transport electrons, were closely correlated to that of Ф_PSII_, implying that the regulation of CO_2_ assimilation rate and PSII electron transport by BR is most likely attributed to the demand for NADPH and ATP (Fig. 3d).

Next, we independently determined the in vivo carboxylation efficiency of RuBisCO (*V*_c,max_) and regeneration rate of RuBP (*J*_max_) by fitting the A/C_i_ curve using Farquhar’s model. The results showed that defects in BR biosynthesis inhibited the *V*_c,max_ and *J*_max_ in *d*^*im*^, whereas *Dwarf* overexpression significantly increased *V*_c,max_ and *J*_max_ as compared to WT (Fig. 3e and f).

### BR promoted the activity of Calvin cycle enzymes

**Fig. 4:**
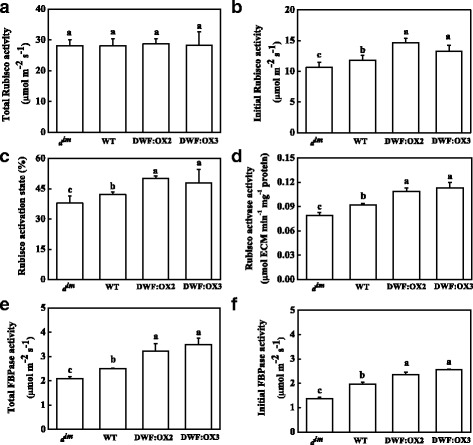
Effects of *Dwarf* gene overexpression (DWF:OX2 and DWF:OX3) and mutation (*d* ^*im*^) on total and initial carboxylation activity of RuBisCO (**a** and **b**), the activation status of RuBisCO (**c**), the activity of RuBisCO activase (**d**), and total and initial activity of fructose 1,6-bisphosphatase (FBPase) (**e** and **f**). Data are the means of four replicates with SDs. Means followed by the same letter are not significantly different according to Tukey’s test (*P* < 0.05)

To get a better insight into the mechanism of BR-regulated photosynthesis, we analyzed the enzymatic activity of RuBisCO and fructose 1,6-bisphosphatase (FBPase), which is critical for the regeneration of RuBP. The results showed that changes in endogenous BR levels had no effects on the total activity of RuBisCO (Fig. 4a). However, BR levels regulated the initial activity of RuBisCO in a similar manner as *V*_c,max_ in different genotypes, leading to an increased and decreased activation state of RuBisCO in the transgenic lines and *d*^*im*^ mutant, respectively (Fig. 4b and c). Consistent with the activation state of RuBisCO, RCA activity was inhibited in *d*^*im*^, and was upregulated in the transgenic lines (Fig. 4d). In contrast to the RuBisCO, BR deficiency in *d*^*im*^ significantly inhibited the total activity of FBPase, whereas the high BR levels in the transgenic lines resulted in significant increases of FBPase activity. Furthermore, the initial activity of FBPase was regulated by BR levels in a similar way (Fig. 4e and f).

**Fig. 5:**
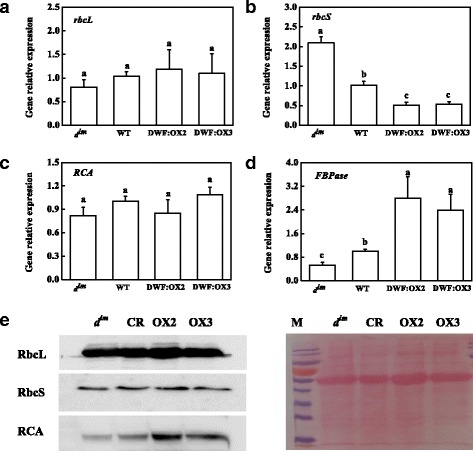
Effects of *Dwarf* gene overexpression (DWF:OX2 and DWF:OX3) and mutation (*d* ^*im*^) on the relative transcript levels of genes encoding RuBisCO large (*rbcL*) and small (*rbcS*) subunit (**a** and **b**), RuBisCO activase (*RCA*) (**c**) and fructose 1,6-bisphosphatase (*FBPase*) (**d**). Data are the means of four replicates with SDs. Means followed by the same letter are not significantly different according to Tukey’s test (*P* < 0.05). Effects on the protein levels of rbcL, rbcS and RCA as detected by western blot were also shown (**e**)

Analysis of gene expression of Calvin cycle enzymes showed that the steady state mRNA levels of *rbcL*, *rbcS* and *RCA*, which encode RuBisCO large subunit, small subunit and activase, respectively, were not consistent with the enzyme activity (Fig. 5a–d). Endogenous BR levels did not affect the mRNA level of *rbcL* and *RCA*. In contrast to the Rubisco activity, BR deficiency in *d*^*im*^ resulted in upregulation of *rbcS*, whereas a high BR level in the transgenic lines inhibited the expression of *rbcS*. Interestingly, the mRNA level of FBPase was positively related to the enzymatic activity, suggesting that BR regulates FBPase at transcriptional level.

Protein content of rbcL, rbcS and RCA as detected by western blot showed a different pattern as transcript levels. Consistent with the total activity of RuBisCO, endogenous BR levels had no effects on the protein level of rbcL and rbcS (Fig. 5e). However, BR deficiency in *d*^*im*^ and overexpression of *Dwarf* resulted in a decrease and increase in the protein content of RCA, respectively.

### BR induced a reduced redox status

**Fig. 6:**
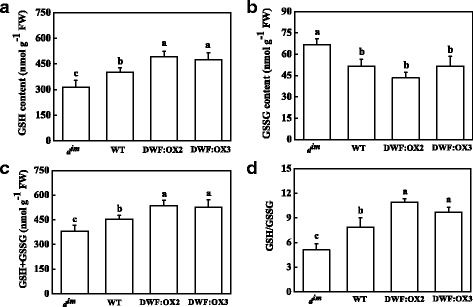
Effects of *Dwarf* gene overexpression (DWF:OX2 and DWF:OX3) and mutation (*d* ^*im*^) on the content of reduced (GSH), oxidized (GSSG), total (GSH + GSSG) glutathione (**a**-**c**) and ratio of GSH/GSSG (**d**). Data are the means of four replicates with SDs. Means followed by the same letter are not significantly different according to Tukey’s test (*P* < 0.05)

**Fig. 7:**
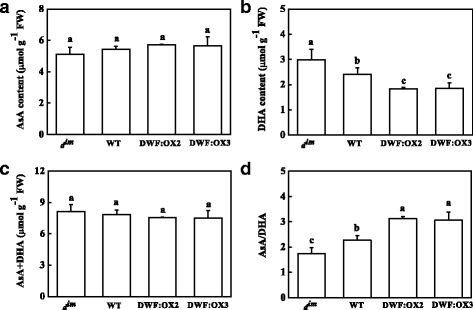
Effects of *Dwarf* gene overexpression (DWF:OX2 and DWF:OX3) and mutation (*d* ^*im*^) on the content of reduced (AsA), oxidized (DHA), total (AsA + DHA) ascorbate (**a**-**c**) and ratio of AsA/DHA (**d**). Data are the means of four replicates with SDs. Means followed by the same letter are not significantly different according to Tukey’s test (*P* < 0.05)

Calvin cycle enzymes are well known to be regulated through redox posttranslational modifications. To further study the mechanism by which BR regulates the activity of RuBisCO and RCA, we analyzed the content and redox status of glutathione and ascorbate. The results indicated that low BR levels in *d*^*im*^ mutant resulted in a decrease in the content of reduced glutathione (GSH), whereas high BR levels in the transgenic lines led to increased accumulation of GSH as compared to WT (Fig. 6a). In contrast, the content of oxidized glutathione (GSSG) was significantly increased in *d*^*im*^, but was not affected in the transgenic lines (Fig. 6b). Total glutathione (GSH + GSSG) showed similar changes as that of GSH (Fig. 6c). Importantly, BR deficiency in *d*^*im*^ led to an oxidized redox status of glutathione, as shown by a decline in the GSH/GSSG ratio, whereas high BR level in the transgenic lines resulted in a reduced redox status of glutathione, as shown by a increase in the GSH/GSSG ratio (Fig. 6d). Different BR levels did not affect the content of reduced ascorbate (AsA) (Fig. 7a). However, BR deficiency significantly increased the content of oxidized ascorbate (DHA), whereas high BR levels led to a significant decrease in DHA content (Fig. 7b). Accordingly, low and high BR levels resulted in an increase and decrease in AsA/DHA ratio in *d*^*im*^ and transgenic lines, respectively (Fig. 7d).

### BR activated the antioxidant enzymes

**Fig. 8:**
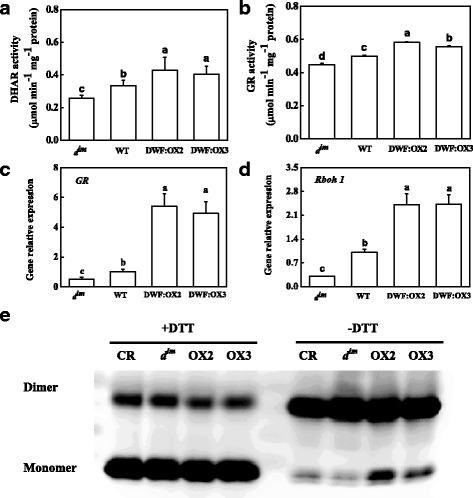
Effects of *Dwarf* gene overexpression (OX2 and OX3) and mutation (*d* ^*im*^) on the activity of dehydroascorbate reductase (DHAR), (**a**), glutathione reductase (GR), (**b**), the relative mRNA abundance of *GR* and *Rboh1* gene (**c** and **d**) and the redox status of 2-cysteine peroxiredoxin (2-CP), (**e**), which was determined by western blot of 2-CP in reducing and nonreducing conditions. The columns represent the means of four replicates with SDs. Means followed by the same letter are not significantly different according to Tukey’s test (*P* < 0.05)

High turnover rate of ascorbate-glutathione cycle is required to maintain the cellular redox status. Assay of dehydroascorbate reductase (DHAR) and glutathione reductase (GR), two key enzymes in the cycle, showed that the activities of DHAR and GR were inhibited in *d*^*im*^ mutant, but were induced by a high BR level in the transgenic lines (Fig. 8a and b). The transcript abundance of *GR* was consistent with the activity of GR (Fig. 8c). Interestingly, the expression of *Rboh1*, encoding the plasma membrane NADPH oxidase, which plays a critical role in the regulation of antioxidant signaling, was inhibited in *d*^*im*^ mutant, but was significantly upregulated in the transgenic lines (Fig. 8d).

Although BR levels in different genotypes dramatically affected the redox status, total content of 2-cysteine peroxiredoxin (2-CP) remained unaltered. Western blot showed that when the protein extract was treated with DTT, most of the 2-CP were in the active monomer/reduced form, and no significant changes in 2-CP protein content were detected among the genotypes (Fig. 8e). However, in conditions without DTT, most of the 2-CP were in the dimer form. BR deficiency in *d*^*im*^ resulted in a slight decrease in monomer of 2-CP, whereas a high level of BR in the transgenic plants induced a remarkable increase in monomeric 2-CP as compared with WT.

## Discussion

BRs stimulate a broad spectrum of biological processes and are able to increase not only the yield but also the quality of the crops [12, 13]. Therefore, manipulation of BR biosynthesis is considered as a promising biotechnological target for improving agricultural production [35]. There are a number of studies on the genetic manipulation of BR biosynthesis, focusing on yield improvement in rice either through modifying the plant architecture [36] or by increasing the CO_2_ assimilation [15]. However, few studies report the genetic modification of horticultural crops by altering the BR biosynthesis. Here, we showed that the stimulation of growth by a high endogenous BR level in the transgenic lines is associated with the promotion of photosynthetic capacity. In contrast, BR deficiency in the BR biosynthetic mutant *d*^*im*^ resulted in a decline in photosynthetic capacity, thus confirming the role of BRs in the regulation of photosynthesis.

From the gas exchange analysis, it is clear that a high BR levels promotes photosynthesis mainly through regulating the nonstomatal factors in the transgenic plants (Fig. 2). However, the reduction in photosynthetic rate in *d*^*im*^ mutant was accompanied with the reduced stomatal conductance and intercellular CO_2_ concentration (Fig. 2), indicating that the CO_2_ diffusion through stomata is at least one of the targets through which BR regulates photosynthetic rate. Our recent study showed that application of a low concentration of BR promotes stomatal opening in tomato [37]. However, we did not observe increased stomatal conductance in the transgenic plants. The discrepancy can be explained by the fact that the influence of CO_2_ on the stomatal aperture was minimized in the in vitro epidermal strip assay, whereas the high level of intercellular CO_2_ concentration (Fig. 2) in the transgenic line may inhibit the stomatal opening [38]. Alternatively, a high BR level may lead to a decline in stomata number [39].

BR regulates the thylakoid structure and function of PSII in Arabidopsis and mutants deficient in BR biosynthesis exhibit a thermal instability of oxygen evolution complex [40]. This explains why the PSII of tomato *d*^*im*^ mutant is more susceptible to photoinhibition during heat stress as compared to WT [41]. However, we did not observe significant difference in Fv/Fm and Fv’/Fm’ (Fig. 3), implying that the intrinsic function of PSII is not affected among the genotypes at least in the present experimental conditions. Given that the thylakoid architecture and PSII function can be maintained in a certain range of BR levels [40], we could not exclude the possibility that maximum quantum yield will be affected when the BR levels is further decreased than *d*^*im*^. In contrast to Fv/Fm and Fv’/Fm’, Ф_PSII_ and qP exhibited consistent changes with that of photosynthetic rate (Fig. 3), suggesting that the photosynthetic electron transport was restricted at sites downstream of PSII, e.g. the electron sink determined by the turnover of NADPH/ATP synthesis and consumption in the dark reaction [42].

Carboxylation and regeneration of RuBP consume substantial amount of NADPH and ATP. A/Ci curve and biochemical analysis of RuBisCO and FBPase provide evidences that BR promotes photosynthesis by positively regulating Calvin cycle enzymes (Figs. 3 and 4). BR regulates the activity of RuBisCO mainly through increasing the activation state but not the total activity of RuBisCO. This coupled with the protein content of rbcL and rbcS as determined by western blot (Fig. 5e) suggested that overexpression or mutation of *Dwarf* had no effects on the RuBisCO content. RuBisCO holoenzyme is composed of small and large subunits, which are encoded by *rbcS* and *rbcL*. Protein synthesis of rbcS and rbcL is tightly coordinated in order to make sure assembly in defined stoichiometric ratios [43]. By contrast, transcript levels of *rbcS* and *rbcL* are not always correlated [43, 44]. Accordingly, BR negatively regulates the transcription of *rbcS* while had no effects on the transcripts of *rbcL* in this study (Fig. 5). Intriguingly, transcriptional upregulation or downregulatino in *d*^*im*^ mutant and *Dwarf*-overexpressing plants did not lead to changes in RuBisCO content. One possible explanation is that the synthesis efficiency of rbcS is different as observed by a previous study where overexpression of *rbcS* did not result in consistent higher RuBisCO content throughout the plant canopy [45]. Alternatively, translation of *rbcL* is limiting for RuBisCO assembly. When cellular glutathione pools are in the oxidized state, a putative repressor motif in rbcL proteins is exposed, leading to binding and translation arrest of *rbcL* mRNA independent of rbcS [46, 47]. Considering the role of BR in regulating the redox status of glutathione (Fig. 6), it is conceivable that the translation of *rbcL* is affected. In contrast to RuBisCO, our data suggest that BR regulates FBPase at transcriptional level (Fig. 5). Knockout of both chloroplastic and cytosolic FBPase results in dwarfism and imbalance in carbohydrate metabolism in Arabidopsis [48]; the phenotypes reminiscent of the BR biosynthetic mutants suggests a close relationship between FBPase activity and BR-regulated developmental processes.

As for the mechanism by which BR positively regulates the activation state of RuBisCO, the glutathione redox status may be involved. GSH plays a role in activation of RuBisCO via promoting the thiol/disulfide exchanges [49, 50]. BR-regulated activity of RuBisCO was associated with a high ratio of GSH/GSSG and AsA/DHA in *Dwarf* overexpressing lines (Figs. 6 and 7). From our results, it is clear that the high GSH/GSSG and AsA/DHA ratios are attributed to the increased activity of DHAR and GR (Fig. 8). Acceleration of GSH-AsA cycle in *Dwarf*-overexpressing lines helps the chloroplast to maintain a reducing state, which facilitates the activation of RuBisCO. BR may activate RuBisCO via redox regulation of RCA [20, 51]. This is supported by the observation that high endogenous BR levels promoted RCA activity and protein content, which is associated with reducing status of glutathione, ascorbate and 2-CP in this study. The reduction of 2-CP is mediated by thioredoxin (Trx)-mediated redox system [52]. Together with the observation of our previous study that BR induces apoplastic ROS [37, 53], upregulation of *Rboh1* by endogenous BRs in the current study (Fig. 8) suggests that BR regulates the whole redox system of the plant, including Trx. It is well established that Trx is critical for activating RCA [54, 55]. Therefore, Trx may play a role in activation of RCA by BR. Indeed, silencing of chloroplast Trx in tomato compromised BR-promoted photosynthesis [28].

## Conclusions

In summary, results of the current study showed that increasing endogenous BR levels by overexpresion of BR biosynthetic gene enhanced quantum yield of photosystem II and CO_2_ assimilation rate. Endogenous BR regulated photosynthetic capacity mainly through activating the activity of Calvin cycle enzymes. BR-inducible ROS signaling may be involved in the redox regulation of Calvin cycle enzymes.

## Additional file

Additional file 1: Table S1. Gene-specific primers designed for qRT-PCR. (DOC 75 kb)

## Abbreviations

2-CP: 2-cystein peroxiredoxin
ABA: abscisic acid
AsA: reduced ascorbate
BRs: brassinosteorids
C_i_: intercellular CO_2_ concentration
DHA: oxidized ascorbate
DHAR: dehydroascorbate reductase
FBPase: fructose 1,6-bisphosphatase
Fv/Fm: maximum quantum yield of PSII
Fv’/Fm’: antenna excitation transfer efficiency
GAs: gibberellins
GR: glutathione reductase
g_s_: stomatal conductance
GSH: reduced glutathione
GSSG: oxidized glutathione
*J*_max_: maximum RuBP regeneration rates
P_N_: net photosynthetic rates
PSII: photosystem II
qP: photochemical quenching coefficient
*rbcL*: RuBisCO large subunit
*rbcS*: RuBisCO small subunit
*RCA*: RuBisCO activase
RuBisCO: ribulose-1,5-bisphosphate carboxylase/oxygenase
RuBP: ribulose-1,5-bisphosphate
Tr: transpiration rate
*V*_c,max_: maximum RuBisCO carboxylation rates
Ф_PSII_: actual quantum yield of PSII
