# Supplementary material for: Overexpression of a brassinosteroid biosynthetic gene Dwarf enhances photosynthetic capacity through activation of Calvin cycle enzymes in tomato
Source: BMC Plant Biol. 2016 Jan 28;16:33. doi: 10.1186/s12870-016-0715-6 (PMC4730719; doi:10.1186/s12870-016-0715-6)
Supplement: Additional file 1: Table S1. — Gene-specific primers designed for qRT-PCR. (DOC 75 kb) [file 12870_2016_715_MOESM1_ESM.doc]

**Supplemental**

**Supplemental Table S1. Gene-specific primers designed for qRT-PCR.**

| Gene | Accession Numbers | Forward primer | Reverse primer |
| --- | --- | --- | --- |
| *Actin* | Solyc11g005330 | TGTCCCTATTTACGAGGGTTATGC | CAGTTAAATCACGACCAGCAAGAT |
| *DWARF* | Solyc02g089160 | TCCTGATCCATATTCGTTCAA | ACCAAGTTCCTTTCCAGGAC |
| *RCA* | Solyc110g086580 | CTGTTGGTCATCCGATGTGT | CCCAAGGTTTCAAACAGGAA |
| *rbcL* | Solyc101g007330 | ACCGCAAATACTACCTTGGC | CCACCAGACATACGTAACGC |
| *rbcS* | Solyc102g063150 | TTGCTTGGAATTCGAGACTG | CTCTTGAACCTCAGCCAACA |
| *FBPase* | Solyc102g062340 | GAAGAGAAATGGCATCAGCA | AGTGAGTCCAGAAGGATGGG |
| *Rboh1* | Solyc08g081690 | TCCAGCACAAGATTACCG | CCTCCATTGCGACGAT |
| *GR* | NM001247314 | TTGGATGGAACTATGAGGCA | CAGAGTGACATCCGCATTCT |
